# Supplementary figures and images for: HIV/AIDS burden, attributable risk factors, and projections among reproductive-age adults in China, 1990–2035: A GBD 2023 analysis
Source: PLoS One. 2026 May 27;21(5):e0350196. doi: 10.1371/journal.pone.0350196 (PMC13215530; doi:10.1371/journal.pone.0350196)

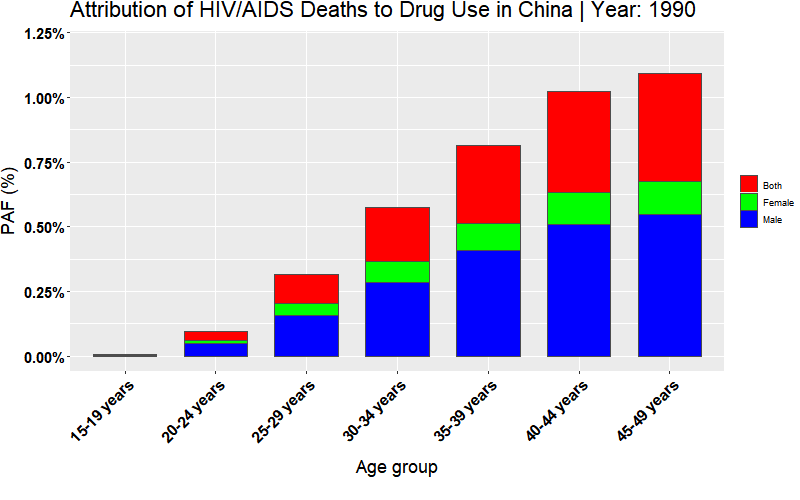

Supplement: S1 Fig — (GIF) [file pone.0350196.s005.gif]

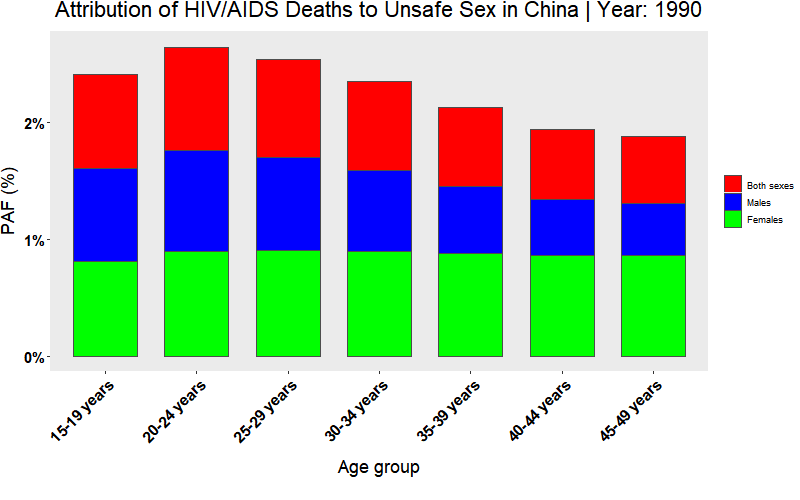

Supplement: S2 Fig — (GIF) [file pone.0350196.s006.gif]

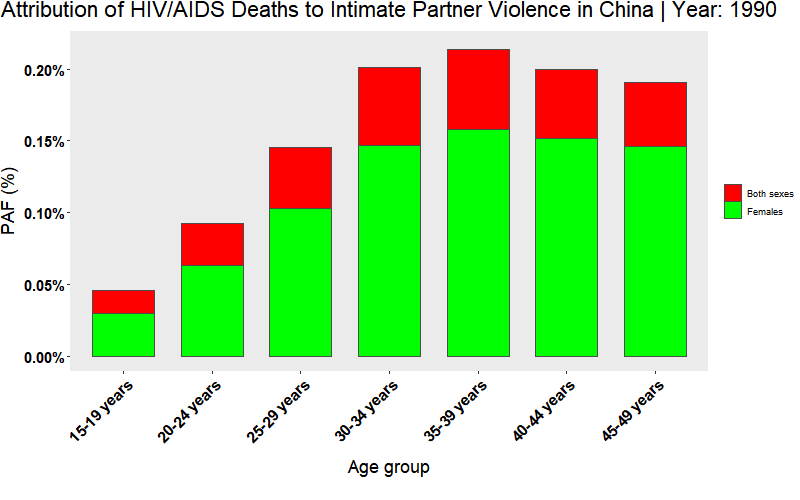

Supplement: S3 Fig — (GIF) [file pone.0350196.s007.gif]

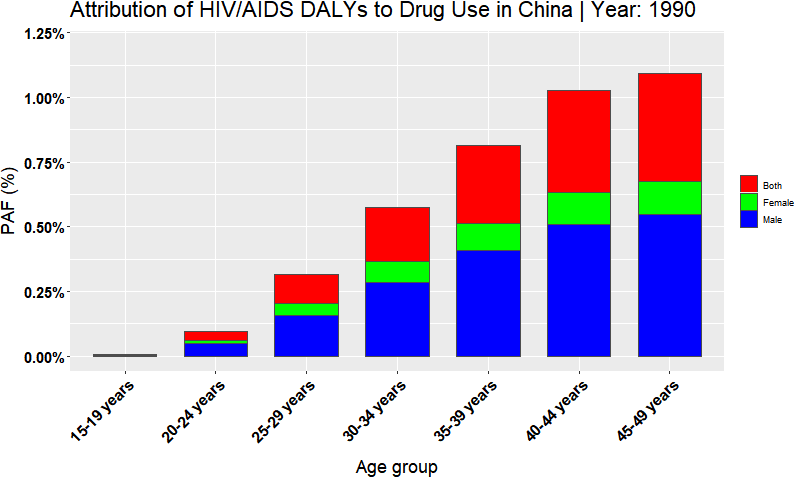

Supplement: S4 Fig — (GIF) [file pone.0350196.s008.gif]

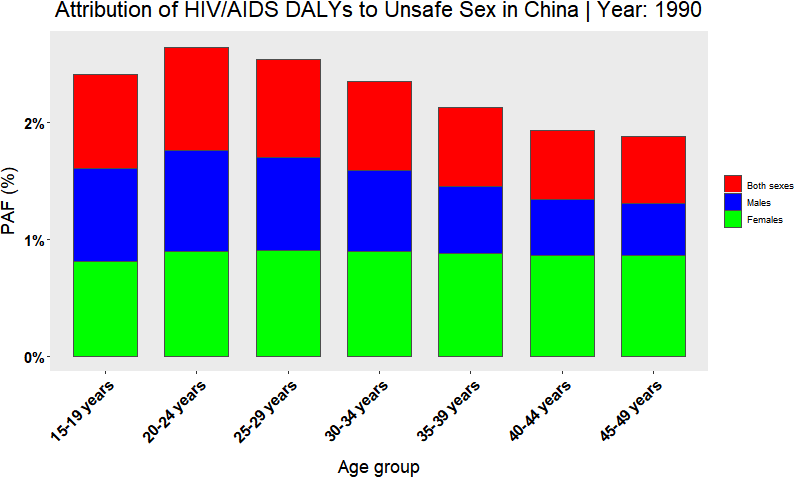

Supplement: S5 Fig — (GIF) [file pone.0350196.s009.gif]

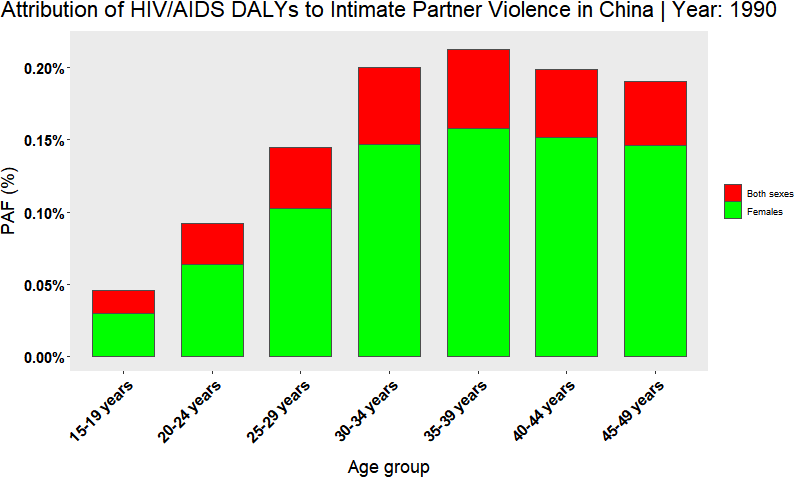

Supplement: S6 Fig — (GIF) [file pone.0350196.s010.gif]
