# Supplementary material for: HIV/AIDS burden, attributable risk factors, and projections among reproductive-age adults in China, 1990–2035: A GBD 2023 analysis
Source: PLoS One. 2026 May 27;21(5):e0350196. doi: 10.1371/journal.pone.0350196 (PMC13215530; doi:10.1371/journal.pone.0350196)
Supplement: Attachment — Submitted filename: Review Comments.docx [file pone.0350196.s011.docx]

This manuscript analyzes the burden, attributable risk factors, and projected trends of HIV/AIDS among reproductive-age adults (15–49 years) in China using Global Burden of Disease (GBD) 2023 data, applying Joinpoint regression and ARIMA forecasting models. The topic is relevant for epidemiological surveillance and health policy planning, and the use of standardized GBD estimates allows for long-term trend assessment.

Overall, the study provides a useful descriptive overview of HIV burden trends in China. However, several issues related to clarity, methodological description, interpretation of results, and manuscript structure should be addressed before the manuscript can be considered for publication. In particular, the introduction does not clearly articulate the knowledge gap, parts of the methods section require clarification, and some interpretations in the discussion appear overly strong given the ecological nature of the data.

**Major comments**

**1. Introduction – clarity of the research gap**

The introduction provides a broad overview of HIV/AIDS epidemiology globally and in China, but the specific research gap addressed by this study is not clearly articulated.

While the manuscript states that this is the “first comprehensive analysis” of HIV burden in reproductive-age adults using GBD 2023 data, this claim should be better justified or moderated. Previous studies using earlier GBD datasets have already examined HIV burden trends in China and globally.

The authors should more clearly explain:

- Why focusing specifically on the **15–49 age group** provides additional insight beyond existing studies.
- What **new contribution** the use of GBD 2023 data provides compared with GBD 2021 or earlier analyses.
- How this study advances the current literature on HIV epidemiology in China.

**2. Methods – clarification of study design**

The study relies entirely on secondary data from the Global Burden of Disease database. However, the manuscript does not explicitly state the study design.

The authors should clarify early in the methods section that this is a **secondary ecological analysis of publicly available GBD data**.

Additionally, more detail should be provided on:

- The exact **data extraction procedures** from the GBD platform.
- Whether uncertainty intervals from the GBD estimates were incorporated into trend analyses.
- The rationale for restricting the ARIMA incidence model to the **2000–2023 time series**.

**3. Selection of risk factors**

The study examines three risk factors: unsafe sex, drug use, and intimate partner violence. However, the process by which these factors were selected requires further clarification.

The authors state that the selection followed the GBD risk factor hierarchy, but it remains unclear:

- Whether other risk factors were evaluated and subsequently excluded.
- Whether the selected risk factors represent the **full set of attributable factors available in the GBD framework for HIV/AIDS**.

This should be clarified to improve methodological transparency.

**4. Interpretation of large percentage increases**

The manuscript reports very large percentage increases in incidence, prevalence, mortality, and DALYs between 1990 and 2023.

While mathematically correct, these large percentage increases may largely reflect **very low baseline rates in the early years of the epidemic**. This point should be explicitly acknowledged in the Results or Discussion sections to avoid misleading interpretation.

**5. Interpretation of causal mechanisms**

Some statements in the discussion imply causal explanations (for example, linking male predominance directly to MSM transmission patterns and drug use). While these explanations are plausible, the analysis is based on **aggregated ecological data**, and therefore causal inference should be interpreted cautiously.

The authors should moderate language suggesting causal mechanisms and clearly distinguish between **observed associations and hypothesized explanations**.

**6. Forecasting analysis**

The ARIMA-based projections to 2035 are a valuable component of the study. However, additional clarification is needed regarding:

- Model diagnostics beyond the Ljung–Box test.
- How uncertainty intervals were propagated into the projections.
- The limitations of ARIMA models for long-term epidemiological forecasting.

These points should be discussed more explicitly.

**7. Redundancy in results and discussion**

Several sections of the manuscript contain repetitive descriptions of the same findings. For example, the male predominance in HIV burden and the transition toward chronic disease burden are mentioned multiple times across the Results and Discussion sections.

The manuscript would benefit from **condensing repetitive sections and improving narrative flow**.

**Minor comments**

1. There is a typographical error in the section heading:

“Intoduction” should be corrected to “Introduction”.

2. The manuscript should standardize the formatting of units, particularly:
   - “per 100 000”
   - “per 100,000”
3. Some sentences are excessively long and difficult to follow. Language editing by a fluent English speaker would improve readability.
4. The legends for several figures are overly technical and could be simplified to improve accessibility for readers.
5. Table 1 is very dense and may benefit from simplification or splitting into separate tables for incidence/prevalence and mortality/DALYs.

**Comments on figures and tables**

The figures are informative but could be improved for clarity.

- **Figure 1:** Joinpoint plots are useful, but the legend is complex and may be difficult for readers unfamiliar with Joinpoint methodology.
- **Figure 2:** The population pyramid visualization is interesting, but the scaling makes visual comparison between sexes somewhat difficult.
- **Table 1:** The table contains redundant metrics (e.g., rate and ASIR). Consider simplifying the presentation to improve readability.

**Limitations**

The limitations section is appropriate but could be expanded to include:

- potential underreporting or uncertainty in surveillance data incorporated into GBD estimates,
- limitations of ARIMA forecasting for long-term projections,
- the lack of subnational analyses, which may mask regional heterogeneity within China.

**Conclusion**

The conclusion summarizes the findings but could be made more concise. Some statements regarding policy implications appear stronger than what can be directly supported by the ecological data.

The authors should focus the conclusion on the main epidemiological findings and avoid overly prescriptive policy statements.

The manuscript addresses an important public health topic and uses a widely recognized dataset. However, revisions are needed to improve methodological transparency, interpretation of results, and overall clarity of the manuscript.
