# Supplementary material for: HIV/AIDS burden, attributable risk factors, and projections among reproductive-age adults in China, 1990–2035: A GBD 2023 analysis
Source: PLoS One. 2026 May 27;21(5):e0350196. doi: 10.1371/journal.pone.0350196 (PMC13215530; doi:10.1371/journal.pone.0350196)
Supplement: Attachment — Submitted filename: Response to Reviewers.docx [file pone.0350196.s012.docx]

**Reviewer #1**

**[Comment 1] Reviewer #1, Major comment 1: Introduction -- clarity of the research gap**

Response:

We sincerely thank the reviewer for this constructive criticism. We acknowledge that the original manuscript contained an unsustainable claim of being the "first comprehensive analysis" in the Abstract (Background section), and the Introduction failed to clearly articulate the specific research gap. We have thoroughly revised both sections to: (1) remove the "first" claim and replace with "updated comprehensive assessment"; (2) explicitly contrast GBD 2023 with earlier versions (2019, 2021); (3) justify the focus on reproductive-age adults (15–49 years) with quantitative evidence; and (4) specify the methodological advancements in GBD 2023 that enhance estimate reliability.

Changes made:

**-Abstract, Background section, Page 1, Lines 16,18-19:**

- From: "This study provides the first comprehensive analysis of HIV/AIDS burden in this population using the most recent Global Burden of Disease Study 2023 data."

- To: "This study provides an updated comprehensive assessment of HIV/AIDS burden in this population using Global Burden of Disease Study 2023 data, which incorporates methodological refinements and post-pandemic data not available in earlier versions."

**- Introduction, Page 2, Lines 59-60:**

From: "To inform this decision, we analysed 1990–2021 HIV/AIDS burden in China and projected trends to 2031 using the Global Burden of Disease 2021 study[4]."

To: "To inform this decision, we analysed 1990–2023 HIV/AIDS burden in China and projected trends to 2035 using the Global Burden of Disease Study 2023 [5]."

Note: Corrected critical errors in time frame (1990–2021 → 1990–2023), projection endpoint (2031 → 2035), and data source (GBD 2021 → GBD 2023).

**- Introduction, Page 3, Lines 63-73 (revised gap statement):**

- From: "While recent GBD-based analyses have highlighted marked international disparities, a comprehensive, population-level portrait of China's reproductive-age population (15–49 years) remains absent[6–8]."

- To: "Earlier GBD analyses (2019, 2021) have documented national HIV trends in China[6–8], yet three critical gaps persist. First, these studies predate the COVID-19 pandemic and associated disruptions to HIV testing and treatment services, necessitating updated estimates through 2023. Second, they did not specifically focus on reproductive-age adults (15–49 years), a demographic that accounts for the majority of new diagnoses in China—males in this age group alone contributing 79.24% of reported cases in 2022^[9]^, and representing a strategic target for prevention interventions including pre-exposure prophylaxis (PrEP) and harm reduction programs. Third, GBD 2023 incorporates methodological refinements—including updated HIV progression models and expanded data sources from 2020–2023—that yield more precise estimates for recent trends."

**- Introduction, Page 2, Lines 74-81 (added GBD 2023 advancements):**

- Added: "Compared with GBD 2021, GBD 2023 offers three key advancements relevant to HIV burden estimation: updated Cause of Death Ensemble modeling (CODEm) with expanded vital registration data from China's disease surveillance points system; revised risk factor attribution methods incorporating systematic reviews through 2023; and inclusion of post-COVID-19 pandemic data (2020–2023) that capture potential disruptions to HIV testing and treatment services. These improvements yield more precise estimates for the most recent period and enhance the reliability of projections to 2035."

**- Introduction, Page 3, Lines 86-90 (clarified study contribution):**

- Added: "Accordingly, this study provides an updated comprehensive assessment of HIV/AIDS burden, attributable risk factors, and projections specifically among reproductive-age adults in China, leveraging the most recent GBD 2023 methodologies to inform targeted prevention strategies and progress toward Sustainable Development Goal 3.3."

**-Discussion, Page 7,lines 282-284.**

opening paragraph: Changed "the first comprehensive application" to "an updated comprehensive assessment... leveraging the most recent GBD 2023 data" for consistency with revised claims in Abstract and Introduction.

**Reference update associated with this comment:**

[5] Inserted: GBD 2023 Disease and Injury and Risk Factor Collaborators. Burden of 375 diseases and injuries... [Lancet. 2025;406(10513):1873-1922]. This replaces the previous URL citation and provides the formal data source reference.

[9] Added: Hou Y, et al. Comparative analysis of epidemiological features of HIV/AIDS cases aged over and under 50 years old—China, 2010–2022. China CDC Wkly. 2023;5(48):1079–1083. (Supports the 15–49 age group focus with quantitative evidence).

**[Comment 2] Reviewer #1, Major comment 2: Methods -- clarification of study design**

Response:

We appreciate this important clarification. We have explicitly stated the study design as a secondary ecological analysis and added detailed information on data extraction procedures, uncertainty interval handling, and the rationale for the ARIMA time restriction. The revised text clarifies that the restriction to 2000–2023 was necessitated by sex-specific analysis requirements rather than data quality concerns alone.

Changes made:

**• Page 3, lines 95-98.**

2.1 Data sources and study design, opening paragraph: Added explicit statement of study design:

"This study is a secondary ecological analysis of publicly available GBD 2023 data. We examined aggregated national-level estimates of HIV/AIDS burden without direct involvement of human participants."

• **Page 3, lines 106-110.**

2.1 Data sources and study design, data extraction paragraph: Reorganized with complete parameter selection details and extraction timeframe:

"We selected the following parameters: Location: China; Cause: HIV/AIDS; Metric: Number, Rate, and Percent; Age: 15–19, 20–24, …, 45–49 years (five-year age groups); Sex: Male, Female, Both; Year: 1990–2023; Measure: Incidence, Prevalence, Mortality, and DALYs. Data were downloaded as CSV files in December 2025 and imported into R 4.3.2 for analysis."

**• Page 3, lines 117-120.**

2.1 Data sources and study design, uncertainty interval handling: Clarified differential use of uncertainty intervals:

"We used posterior means and their 95 % uncertainty intervals to derive standard errors for joinpoint regression (SE = [upper − lower]/3.92) as required by Joinpoint 5.2 software; ARIMA models used posterior mean estimates as point inputs without uncertainty weighting, with prediction intervals derived from the model's error variance."

**• Page 4, lines 153-159.**

2.2 Statistical Methods, ARIMA section: Clarified rationale for time restriction and added reference to sensitivity analyses:

"Because sex-specific ASIR series failed white-noise diagnostics for 1990–2023 (Ljung-Box p < 0.05), male and female ASIR were separately modelled on 2000–2023 data, while ASPR, ASMR and ASDR used the full 1990–2023 series. This approach balances statistical model requirements with maximum use of available data; joinpoint sensitivity analyses confirmed robustness of trend estimates to alternative model specifications and time-window restrictions (Supplementary Tables S2–S3)."

**[Comment 3] Reviewer #1, Major comment 3: Selection of risk factors**

Response:

We appreciate this request for clarification. We have expanded the description of risk factor selection to explicitly state that four behavioral risk factors were evaluated, one was excluded due to unstable estimates, and the remaining three represent the complete set of robust attributable factors available in the GBD 2023 framework for HIV/AIDS in China.

Changes made:

**• Page 5, lines 169-171, 173-179.**

Methods, Risk factor analysis section: Revised to clarify evaluation and exclusion process:

"HIV/AIDS attributable deaths and DALYs were analysed for four behavioral risk factors: unsafe sex, drug use, intimate-partner violence, and sexual violence against children and bullying. Factor selection followed the GBD 2021 Scientific Council criteria ^[10]^ and Lu et al.'s global analysis ^[6]^. Within the GBD three-tier hierarchy we evaluated all Level 2 behavioral risks available for China; sexual violence against children and bullying was excluded because its 95% uncertainty interval included negative values (lower bound < 0), indicating unstable estimates inconsistent with the counterfactual causal framework. Environmental and metabolic risks were also excluded because no attributable burden estimates were available for HIV/AIDS in China. We retained unsafe sex, drug use, and intimate-partner violence as the three risk factors with robust, non-crossing attributable burden estimates for China."

**[Comment 4] Reviewer #1, Major comment 4: Interpretation of large percentage increases**

Response:

We appreciate this important clarification. We have added explicit caveats in the Results and Discussion sections to contextualise the large percentage increases and avoid misleading interpretation.

Changes made:

**• Results 3.1, lines 197-203.**

burden trends paragraph: Added baseline context and recent trend clarification:

"These large percentage increases reflect the very low baseline rates in 1990, when the HIV epidemic was in its early stages in China. Notably, age-standardised incidence peaked around 2003–2004 and has since declined or plateaued (Figure 1A), indicating that the epidemic is no longer in a phase of rapid expansion. The continued rises in prevalence, mortality, and DALYs reflect improved survival on antiretroviral therapy rather than escalating transmission."

**• Discussion, lines 316-317, 320-322.**

policy landscape paragraph: Embedded interpretive context within existing analysis:

"The steep rise in incidence from 1990 to 2004 reflects limited public knowledge and scarce prevention services in the early epidemic phase^[1]^, occurring from an extremely low baseline when HIV was first emerging in China. [...] While the large percentage increases in cumulative burden between 1990 and 2023 appear substantial, they primarily reflect this low baseline and early expansion rather than recent epidemic growth. The continued rise in prevalence-related mortality and DALYs since incidence plateaued underscores a critical challenge: the growing chronic-care demand from an ageing, treatment-experienced population^[2]^."

**[Comment 5] Reviewer #1, Major comment 5: Interpretation of causal mechanisms**

Response:

We appreciate this important reminder. We have moderated causal language in the Discussion to distinguish observed associations from hypothesized explanations, consistent with the ecological nature of the data.

Changes made:

**• Discussion, lines 288-289.**

opening paragraph: Softened the explicitly flagged causal attribution:

"(ii) males bear disproportionately higher burden across all metrics, consistent with higher reported rates of MSM transmission and drug use"

**• Discussion, line 335.**

male predominance paragraph: Moderated inferential claim:

"may indicate gaps in test-and-treat and PrEP coverage" (replacing "suggests")

**• Discussion, lines 366-369.**

Limitations, opening paragraph: Added explicit ecological inference caveat:

"First, these interpretations are based on ecological associations and should be considered hypothesis-generating rather than definitive causal inferences. Individual-level data on transmission routes and risk behaviors were not available in the GBD aggregated estimates."

**[Comment 6] Reviewer #1, Major comment 6: Forecasting analysis**

Response:

We appreciate the opportunity to clarify the ARIMA methodology. We have expanded the description of model diagnostics, uncertainty interval derivation, and long-term forecasting limitations.

Changes made:

**• Methods, lines 159-163.**

ARIMA section: Expanded model diagnostics description:

"Model diagnostics included the Ljung-Box test for residual white noise and inspection of residual autocorrelation functions. All final models showed no significant residual autocorrelation (Ljung-Box p > 0.05, Table S1) and were selected based on the lowest corrected Akaike information criterion (AICc)."

**• Methods, lines 165-168.**

ARIMA section: Clarified uncertainty interval propagation:

"Ninety-five percent prediction intervals were derived from the ARIMA model's error variance using the forecast package's analytical formulae; these intervals reflect model-based uncertainty and do not incorporate additional uncertainty from the GBD input estimates."

**• Discussion, lines 380-383.**

Limitations, fourth point: Expanded ARIMA limitations:

"Fourth, ARIMA projections assume continuity of historical trends and do not account for external shocks such as policy changes or behavioral shifts. Prediction intervals substantially widen with forecast horizon, so we have limited primary projections to 2030–2035 to minimize long-term extrapolation error."

**[Comment 7] Reviewer #1, Major comment 7: Redundancy in results and discussion**

Response:

We appreciate this observation. We have identified and removed redundant descriptions, particularly the duplicated methodological discussion, and streamlined references to the analytical framework across the Discussion and Conclusion.

Changes made:

•Discussion: Deleted paragraph 6, which duplicated the methodological description in paragraph 5. The consolidated paragraph now provides a single, coherent description of the Joinpoint-ARIMA framework.

**• Discussion, lines 296-297.**

opening paragraph: Simplified reference to analytical methods to avoid repetition with later detailed description:

> "...while this analytical approach is readily transferable to other middle-income countries facing similar epidemiological transitions^[3,14]^."

**• Discussion>Conclusion: lines 402-404.**

Simplified reference to analytical framework:

> "This analytical framework may be applicable to other middle-income countries facing similar epidemiological transitions. It supports evidence-based public health decision-making in diverse settings."

**• Results 3.3: line 237.**

Simplified concluding sentence to improve narrative flow:

> "...approximately four times the female peak of 7,000 person-years (Figure 2D), with rate patterns detailed in section 3.4."

**Response to Minor Comments**

**[Minor comment 1]** There is a typographical error in the section heading: "Intoduction" should be corrected to "Introduction".

Response:

We thank the reviewer for catching this error. We have corrected the spelling to "Introduction" in the revised manuscript.

**[Minor comment 2]** The manuscript should standardize the formatting of units, particularly: "per 100 000" vs "per 100,000".

Response:

We have standardized all unit formatting to "per 100,000" (comma-separated) throughout the manuscript text, tables, and figure legends for consistency. Specifically:

All numerical values in the text now use comma separators (e.g., 32,372 rather than 32 372).

Figure 3 has been updated with the Y-axis labels revised to "(per 100,000)" for incidence, prevalence, deaths, and DALYs rates.

The updated high-resolution figure file has been re-uploaded to the submission system.

**[Minor comment 3]** Some sentences are excessively long and difficult to follow. Language editing by a fluent English speaker would improve readability.

Response:

We appreciate this suggestion. We have thoroughly revised the manuscript to improve readability by systematically breaking down overly long sentences throughout the text, particularly in the Introduction and Discussion sections. For example:

The concluding paragraph has been restructured into shorter, clearer statements.

Complex nested clauses have been separated into independent sentences.

The revised manuscript has been carefully reviewed by the co-authors to ensure fluency and clarity.

**[Minor comment 4]** The legends for several figures are overly technical and could be simplified to improve accessibility for readers.

Response:

We appreciate this suggestion. After careful consideration, we have retained the detailed APC (Annual Percentage Change) values in the Figure 1 legend to ensure immediate interpretability without requiring readers to cross-reference supplementary materials. We believe this enhances accessibility for the target audience (epidemiologists and public health practitioners) who require precise quantitative trend information.

Changes made:

Figure 3: Standardized unit format to "(per 100,000)" for consistency with manuscript text (see Minor comment 2).

Figures 2, 4, 5, and 6: Retained with existing clear legends; no simplification was necessary as these follow standard epidemiological reporting formats.

**[Minor comment 5]** Table 1 is very dense and may benefit from simplification or splitting into separate tables for incidence/prevalence and mortality/DALYs.

Response:

We have split the original dense Table 1 into two separate tables as suggested:

Table 1: HIV/AIDS Incidence and Prevalence among reproductive-age adults (15–49 years) in China, 1990–2023.

Table 2: HIV/AIDS Deaths and DALYs among reproductive-age adults (15–49 years) in China, 1990–2023.

We use "Deaths" rather than "mortality" in the table title to maintain consistency with the GBD 2023 database nomenclature, where the specific metric is coded as "Deaths". This aligns with standardized GBD reporting conventions while addressing the density concern. Each table now presents fewer columns, making the 95% uncertainty intervals and temporal trends easier to interpret.

All in-text citations have been updated accordingly: references to specific metrics now point to the appropriate table (Table 1 for incidence/prevalence, Table 2 for deaths/DALYs), while summary statements citing all burden indicators now refer to Tables 1–2.

**Response to Comments on Figures and Tables**

**Figure 1:** Joinpoint plots are useful, but the legend is complex and may be difficult for readers unfamiliar with Joinpoint methodology.

Response:

We appreciate this comment and have carefully considered simplifying the legend. However, we respectfully argue that retaining the detailed Annual Percentage Change (APC) values in the figure legend is essential for this analysis. The asterisks (*) indicating statistically significant trend changes are integral to interpreting the joinpoint results—they allow readers to immediately distinguish meaningful inflection points from random fluctuation without cross-referencing supplementary materials.

Removing these values would fundamentally compromise the figure's utility, as the key finding is not merely that trends change, but precisely where they change significantly and by what magnitude (e.g., 1990–2004: +22.7%, 2004–2013: -4.5%).

To address accessibility concerns while preserving this critical information, we have:

Added a brief explanatory note: "APC = Annual Percentage Change; * indicates statistically significant trend change (p < 0.05)."

Ensured the legend follows standard epidemiological reporting conventions for joinpoint regression analyses [citations: National Cancer Institute SEER, GBD study methodologies].

We believe this approach best serves the target audience (epidemiologists and public health practitioners) who require precise quantitative trend information for policy evaluation.

**Figure 2:** The population pyramid visualization is interesting, but the scaling makes visual comparison between sexes somewhat difficult.

Response:

We thank the reviewer for this observation. We confirm that Figure 2 intentionally presents absolute numbers to illustrate the actual service delivery burden and resource requirements for each age-sex stratum. This is epidemiologically distinct from Figure 3, which presents crude rates by age group (per 100,000), showing the actual disease occurrence level within each specific age band.

The marked difference in scale between sexes (males accounting for ~75–80% of absolute cases) is itself a critical finding—it visually demonstrates the disproportionate total burden on the healthcare system. To facilitate interpretation while retaining this essential absolute burden information, we have:

Added vertical reference lines at key age groups to aid visual alignment across sexes

Clarified in the legend: "Absolute numbers reflect actual service burden; for crude rates by age group see Figure 3"

This complementary design allows readers to appreciate both the absolute magnitude (Figure 2) for health system resource planning and the age-specific crude rates (Figure 3) for identifying high-risk age bands within each sex.

**Table 1:** The table contains redundant metrics (e.g., rate and ASIR). Consider simplifying the presentation to improve readability.

Response:

We have addressed this by splitting the original table into Table 1 (Incidence/Prevalence) and Table 2 (Deaths/DALYs), significantly reducing column density.

Regarding crude rates versus age-standardized rates (ASR): these serve distinct purposes. Crude rates reflect actual service demand for resource allocation, while ASR enable valid cross-temporal comparisons. Following GBD conventions, we have retained both but improved clarity with explicit column headers and a footnote explaining their distinct utilities.

**Comments on limitations**

The limitations section is appropriate but could be expanded to include: (a) potential underreporting or uncertainty in surveillance data incorporated into GBD estimates; (b) limitations of ARIMA forecasting for long-term projections; (c) the lack of subnational analyses, which may mask regional heterogeneity within China.

Response:

We thank the reviewer for these valuable suggestions. We have substantially expanded the Limitations section to address all three aspects while maintaining conciseness. The revised section now comprises five focused points:

Regarding surveillance data uncertainty (point a):

We have merged this with the existing time-lag limitation into a consolidated second point that addresses GBD data quality comprehensively. This section now explicitly acknowledges potential underreporting biases due to stigma, limited testing coverage, or diagnostic gaps—particularly during the early epidemic period (1990s)—alongside the inherent data release lag (October 2025). We note that while GBD applies spatiotemporal Gaussian process regression and Cause of Death Ensemble modelling (CODEm) to adjust for these biases, residual uncertainty persists and is quantified in our reported 95% uncertainty intervals.

Regarding ARIMA forecasting limitations (point b):

We have strengthened the fourth point to explicitly state that ARIMA models assume temporal stationarity and cannot account for abrupt policy changes or unforeseen epidemiological shifts. We emphasize that prediction intervals widen substantially with forecast horizon, and clarify that we have intentionally limited primary projections to 2030–2035 to minimize long-term extrapolation error. These projections are now explicitly framed as "business-as-usual" scenarios rather than deterministic forecasts.

Regarding subnational heterogeneity (point c):

We have expanded the fifth point to explicitly acknowledge that province-level heterogeneity was not examined and that this aggregation may mask substantial regional variations—such as distinct epidemiological profiles between southwestern provinces (historically driven by injection drug use) versus eastern coastal regions (sexually transmitted patterns). We identify subnational stratification as a priority for subsequent research essential for targeted resource allocation.

These additions strengthen the manuscript's transparency regarding the scope and boundaries of our findings, while reinforcing that these limitations do not substantively alter our primary conclusions regarding sex-specific trends and the chronic disease transition.

**Comments on conclusion**

The conclusion summarizes the findings but could be made more concise. Some statements regarding policy implications appear stronger than what can be directly supported by the ecological data. The authors should focus the conclusion on the main epidemiological findings and avoid overly prescriptive policy statements.

Response:

We thank the reviewer for this valuable guidance. We have carefully revised the Conclusion to address both concerns while maintaining the clear, concise structure we had previously established.

Regarding policy statements:

We have softened the language throughout to ensure policy implications are evidence-based and suggestive rather than prescriptive, appropriately reflecting the limitations of ecological data. Specific revisions include:

Changed "underscore the need to integrate" to "suggest the value of integrating"

Modified "Gender-differentiated strategies are essential" to "with consideration of gender-differentiated strategies"

Replaced "highlight an urgent priority" regarding female burden with "highlight a need for attention"

Changed "require intensification" of PrEP services to "could help address these trends"

Softened "sustained investment... remains essential" to "Maintaining... remains important"

Modified "directly inform" the National Action Plan to "provide evidence for"

Replaced "readily transferable" with "may be applicable to" other countries

Regarding focus:

The Conclusion now maintains strict focus on the main epidemiological findings: (i) the transition to chronic disease burden with sex-specific profiles; (ii) stabilising incidence but rising mortality/disability; (iii) key risk factors (unsafe sex, drug use); and (iv) projected increases in female burden. Policy implications are now framed as potential applications of the evidence rather than direct prescriptions.

We believe these revisions strike an appropriate balance between highlighting the relevance of our findings for public health and respecting the boundaries of ecological data inference.

**Reviewer #2:** The author made a thorough analysis of the data and all are also available and easily accessible in the manuscript. The data in the GBD reference, while you may need to log in or create an account to access it, the site is for free and if we need to refer back to these, it is also accessible for anyone. Statistical analysis is also easy to understand through the data presented and is supported through the discussion part. I also appreciate the dedicated definition of terms used by the author in the manuscript. This makes it easier for the reader to have a reference of all the abbreviations used in the manuscript.

**Response:**

We thank Reviewer #2 for the positive assessment of our manuscript and for confirming the accessibility and transparency of our data sources and analyses.

Regarding data accessibility:

We confirm that all GBD 2023 data used in this study are publicly available via the IHME Visualization Hub (https://vizhub.healthdata.org/gbd-results/) following free registration. We have now formally cited the published GBD 2023 study [5] to ensure readers can access both the original database and the methodological documentation.

Regarding terminology:

We are pleased that the glossary of abbreviations and definitions enhances readability. All abbreviations (ASIR, ASPR, ASMR, ASDR, APC, etc.) are defined at first use in the main text and summarized in the Abbreviations section for quick reference.

We appreciate the recognition of our analytical rigor and the clarity of our statistical presentation. No further changes were requested by this reviewer.
